# A community-level complementary-food safety and hygiene intervention improves family-food preparation behaviours in rural Gambia: a follow-up of a cluster randomised controlled trial

**DOI:** 10.1136/bmjgh-2024-017026

**Published:** 2026-03-18

**Authors:** William E Holdsworth, Buba Manjang, James T Martin, Ellen Harris-Snell, Sandy Cairncross, Francesca L Crowe, Semira Manaseki-Holland

**Affiliations:** 1 College of Medical and Dental Sciences, University of Birmingham, Birmingham, UK; 2 Deputy Director Public Health Services, Directorate of Public Health, Ministry of Health and Social Welfare, Banjul, Gambia; 3 Department of Applied Health Sciences, College of Medicine and Health, University of Birmingham, Birmingham, UK; 4 Department of Disease Control, London School of Hygiene & Tropical Medicine, London, UK

**Keywords:** Hygiene, Public Health, Prevention strategies, Cluster randomized trial

## Abstract

**Introduction:**

Infectious diarrhoea causes millions of deaths annually in low-income countries. Prevention strategies minimising transmission of diarrhoeal pathogens could include adopting better food hygiene practices. The objective was to assess whether a complementary-food hygiene intervention improved family-food hygiene practices in rural Gambian households.

**Methods:**

A parallel cluster randomised controlled trial was conducted in central Gambia. 30 villages were randomised within strata (north/south of the river, population quartiles) to intervention or control (1:1 ratio) by a UK statistician using a computer-generated sequence. Clusters had a population of 200–450, two health workers and were more than 5 km apart. The 4-day community-based intervention occurred over 1 month, with a reminder visit 4 months later. Competitions, performing arts and songs encouraged caregivers to practice five target complementary-food hygiene behaviours using emotional drivers and social norms. Control villages received a 1 day campaign on water usage in gardening. Caregivers lived in the same village during the intervention and had a 6–36 month old child, though some were new mothers. Findings reported here were secondary outcomes analysed as intention-to-treat. This included the proportion of occasions caregivers practiced five food hygiene behaviours for family-food preparation (three handwashing, one dishes/utensils washing and one re-heating food), measured by direct observation 32 months post intervention. Observers did not deliver the intervention and were masked/blinded to the group allocation of the villages.

**Results:**

At 32 months (20 September 2017 to 26 October 2017), 371 and 376 caregivers were analysed from 15 intervention and 15 control villages, respectively. There was greater adherence to the five behaviours in the intervention group; intervention 2073/4425 (47.0%), control 1827/4559 (40.1%), rate ratio (RR) 1.17 (95% CI 1.08 to 1.27, p<0.001), driven by better adherence to handwashing behaviours.

**Conclusion:**

This community-based complementary-food hygiene intervention additionally improved family-food hygiene behaviours 32 months post intervention.

**Trial registration number:**

PACTR201410000859336.

**WHAT IS ALREADY KNOWN ON THIS TOPIC**

- Three studies have demonstrated an improved adherence to complementary-food hygiene behaviours in response to community-based interventions in developing countries.
- However, no randomised controlled trials have assessed the impact of such interventions on adherence to hygiene practices associated with food fed to the wider family.

**WHAT THIS STUDY ADDS**

- This cluster randomised control trial found statistically significant improvements, demonstrable 32 months after intervention, in all three handwashing behaviours associated with family-food handling as an additional benefit of our community-based complementary-food hygiene intervention.

**HOW THIS STUDY MIGHT AFFECT RESEARCH, PRACTICE OR POLICY**

- Interventions grounded in theories of behaviour change and formative research will increase the likelihood of achieving sustained improvements in food hygiene behaviours.
- Future trials must target both complementary and family food, as the latter is frequently fed to children under the age of 5.

## Introduction

 Diarrhoea is an important contributor to morbidity and mortality in older children and adults. In 2016, there were 1.26 billion episodes and over 300 000 deaths from diarrhoeal disease among people over 5 years of age in sub-Saharan Africa.^1^ The WHO advocates a reduction of contamination of food throughout the food chain, especially at the point of consumption, as a means to reduce the worldwide burden of diarrhoea.^2^

The 2015 WHO’s Global Burden of Foodborne Disease report estimates that in 2010, 11 foodborne hazards caused almost 550 million diarrhoeal illnesses and 230 000 deaths globally, with the highest burden occurring in sub-Saharan Africa.^2^ The importance of food safety (free of foodborne diseases caused by microorganisms) has been reiterated by the United Nations as part of Action Track 1 of the Food Systems Summit 2021.^3^ Adopting household-level hygienic practices related to the preparation, cooking and eating of food is one strategy to prevent foodborne transmission of diarrhoeal pathogens.^4^ Relatively few studies from low and low-middle income countries (LMICs) have demonstrated improvements to the practice of appropriate food hygiene behaviours relating to family food (ie, for family members aged 5 years and above) following community-based food hygiene interventions.^5^ Evidence suggests that the majority of household-level food safety and hygiene promotion programmes instead target children’s food and are add-on components of wider water, sanitation and hygiene programmes (addressing other determinants of diarrhoeal illnesses) or child-nutrition programmes with little focus on food safety and hygiene that can enable behaviour change.^6 7^

The majority of household-level food in LMICs, where the highest burden of diarrhoea lies,^1^ is made at home and food prepared for the wider family is often fed to young children.^8^,^13^ Changing routine daily behaviours in the domestic setting in LMICs is fraught with challenges such as kitchen surfaces that are difficult to clean,^8 14^ a lack of fuel for reheating food,^8 15^ unclean drinking water and a heavy female workload.^89 15^,^17^ This is combined with the finding that despite widespread knowledge of ‘Germ Theory’, fear of disease fails to motivate individuals to improve hygiene behaviour in the longer term.^16^ The Evo-Eco approach to behaviour change,^18^ grounded in evolutionary biology and ecological psychology, considers behaviour as an adaptive response to a changing environment mediated via a ‘checklist’ of emotional drivers of behaviour. Hence, factors such as status, nurture, affiliation and social norms may be more important for motivating people to wash their hands with soap^16 17^ and practice food hygiene behaviour.^8^ Community involvement has also been conceived as important to support mothers in their behaviour change.^8 16 17 19^ Therefore, behaviour change interventions that incorporate these components may be more effective than those that only address knowledge exchange or disease concerns^18^ without wider community support. Other behaviour change studies conducted in LMICs have shown that interventions developed using Hazard Analysis Critical Control Point (HACCP) (a system for identifying and reducing the risk of food safety hazards) alone,^20^,^22^ or in combination with the Evo-Eco theory of change,^9 23 24^ led to a reduction in the contamination of complementary foods (ie, food prepared specifically for infants from 6 months of age) and/or an increased adherence to safe complementary-food hygiene practices.

A cluster randomised controlled trial (RCT) assessed the effectiveness of a community-based campaign-style intervention in improving complementary-food hygiene behaviours among caregivers in rural Gambia.^23 24^ This low-cost scalable intervention used HACCP to identify unhygienic practices and correct behaviours to reduce risk, and targeted caregivers’ emotional drivers and social norms. Results at 6 and 32 months after the initial main intervention showed caregiver behaviour change, reductions in microbiological contamination of complementary food and a 60% reduction in mother-reported childhood diarrhoea.^24^ Caregivers also cook food for the wider family; therefore, we hypothesised that the intervention may have additionally changed family-food hygiene behaviours. Such improved behaviours could be important for reducing contamination of the food that other household members, including other young children, consume. We suggest this is equally important for the young child since a resulting reduction in adult diarrhoea could reduce the pool of pathogens to be transmitted to the young child.

This paper presents the results of a cluster RCT conducted in rural Gambia to assess whether the community-level complementary-food hygiene targeted intervention had an effect on secondary outcomes related to family-food hygiene behaviours detectable in the long term at 32 months (no intervention after fifth month). This was an a priori secondary outcome of the 32-month trial follow-up and is reported in accordance with CONSORT (Consolidated Standards of Reporting Trials) 2025 guidance.

## Methods

### Study design and participants

A parallel cluster RCT was conducted in the Central River Region (CRR) of The Gambia. A cluster design was employed as the intervention was to be delivered at the community level. This took place between 16 February 2015 and 28 April 2015 (online supplemental appendix 1). The full trial methods, cluster and participant eligibility criteria and details of the intervention have been reported previously.^23^ The analysis presented here included the same households but used observations based on hygiene behaviours related to family food rather than complementary food. The study was registered with the Pan African Clinical-Trial Registry, number PACTR201410000859336 (https://pactr.samrc.ac.za/TrialDisplay.aspx?TrialID=859) on 22 July 2014.

Eligible caregivers were those with a child aged 6 to 36 months, lived in the study villages at the time of the intervention (though some did not have a child at the time of intervention) and were available to prepare food. A child-health clinic register was used as the sampling frame, from which approximately 26 caregivers per village were randomly selected by the head of the evaluation team. All caregivers gave written and informed consent. In the case of illiteracy, caregivers (or head of the household) were read out the participant information sheet and provided a thumbprint or signature.

### Randomisation and masking

Randomisation, performed by a UK statistician, was stratified by north or south of the Gambia River and quartiles of cluster population size using a computer-generated sequence. At follow-up, the caregivers and behaviour assessors (observers) were newly recruited.

It was not possible to mask members of the community receiving the intervention; however, the observers assessing the outcomes at 32 months were not involved in the delivery of the intervention and they were unaware there had been an intervention/control group or an RCT. Participants and their assessors were informed that the purpose of the evaluation was to assess domestic food and water usage and this was reflected in the questionnaire where questions in the larger evaluation addressing food and water usage acted as a diversion from those addressing trial outcomes. Numerous precautions to minimise observer and reaction bias are described elsewhere (online supplemental appendix 2).^24^

### Procedures

Mixed methods formative research was conducted in three non-study villages to inform contextualisation and intervention development.^8^ Five food and one water preparation step was identified as posing the greatest risk to complementary-food contamination. These were: having unwashed hands before food preparation, contamination of hands during cooking or prior to feeding the child, use of unclean utensils, not reheating child’s food after storage and the lack of available clean water for the child to drink. From these, six corresponding target complementary-food hygiene practices were ascertained: handwashing with soap and water before and during food preparation as well as prior to feeding the child, washing of pots and utensils before preparing food, reheating stored food and boiling water prior to child’s consumption. Nurture, affiliation, status, disgust and attraction were determined as key emotional drivers to motivate handwashing behaviour change (online supplemental appendix 3) using the Evo-Eco model^18^ and as a theoretical basis of behavioural determinants.

The outline of the intervention schedule and activities (online supplemental appendix 4) was designed by a research team at the University of Birmingham, a team of local public health officers (PHOs) and traditional communicators (TCs) (individuals using traditional African drumming, singing, dancing and performing arts to convey health messages) from CRR region in The Gambia. Emphasis was placed on developing a low-cost, scalable and culturally engendered programme that could be implemented by health promotion workers and community members.

The community-level intervention was delivered by a single implementation team of two TCs and three PHOs. All team members were trained to deliver the intervention, were involved in its development and had previous experience of health promotion. Main intervention delivery occurred across 4 days (days 1, 2, 17 and 25), between which older mother volunteers (MaaSupervisors) visited mothers of young children to reinforce the behaviours. A 5-month reminder visit was included in order to help sustain behaviour change. After this, there was no other intervention input in the control or intervention communities.

The intervention was delivered both at a community and household level. Community events were advertised to villagers by TCs playing a campaign song around the village, followed by house-to-house visits encouraging all community members (including fathers and children) to attend the afternoon meeting. This took place at the village ‘Bantaba’ (a central place typically used for village gatherings) and was opened with prayers often led by the local Imam or community leader in order to encourage community ‘buy in’. The focus of these meetings was a fictional mother who acted as a role model for participants. Known as ‘MaaChampion’, this character practised exemplary complementary-food hygiene behaviours out of love for her child, for which the community admired her. The characters were depicted by TCs using performing arts (songs, drama and story-telling) integrating emotional drivers to motivate behaviour change. A ‘competition’ was introduced for caregivers (usually mothers) to pledge to, and then adopt, correct food hygiene behaviours. Successive afternoon community meetings incorporated additional activities specifically targeting handwashing behaviour: a Glo Germ demonstration that showed ‘glowing germs’ on hands washed without soap using ultraviolet light, and the screening of two animations included in a handwashing hygiene trial in India.^17^

Village-wide events were bolstered by morning household visits of pledged MaaChampion mothers by the intervention team and MaaSupervisors to encourage and ascertain uptake of the correct complementary-food hygiene behaviours and provide feedback on their performance. MaaSupervisors also visited these and other mothers with young children as many times as they could between and after the campaign day visits. Given that a condition of being a MaaChampion was to recruit two other mothers, then this peer support and education also occurred between and following campaign days. Caregivers were also supplied with several environmental cues. These included kitchen danglers detailing target hygiene behaviours that were installed at eye level to act as a reminder, in addition to a plastic sheet to help dry pots and utensils on a clean surface once washed. Towards the end of the intervention, successful caregivers who achieved MaaChampion status were given a medal and had their photo placed on a village honour board. A single bar of soap was only provided to caregivers as a gift after they had achieved MaaChampion status.

In place of the intervention, control communities received a 1 day educational campaign on water usage in domestic vegetable gardening during the same time period as the intervention. This was delivered by a PHO to a whole community meeting using flipcharts to ensure that control caregivers had received sufficient attention during the study.

Outcome assessment for preparing complementary-food and family-food practices was conducted 32 months after the beginning of the intervention. 30 female Gambian secondary school graduates were newly recruited from non-study villages and trained as observers within households, with one village being assessed per day. Each caregiver was shadowed between approximately 06:00 and 15:00 by an observer who assessed the caregiver’s adherence to five target family-food preparation related behaviours. Observers also completed a sociodemographic questionnaire according to the caregiver’s responses to questions regarding household characteristics. Presence of soap in the kitchen, latrine and handwashing station was also recorded. Records were checked for completeness and internal consistency at the end of each assessment day.

### Outcomes

The primary outcome was the difference between the intervention and control clusters in key family-food related behaviours, defined as: (1) handwashing before food preparation; (2) handwashing after hand contamination during cooking; (3) handwashing before the caregiver eats; (4) washing of pots and utensils before food preparation or before eating (caregiver) and drying on a clean surface; and (5) reheating of precooked food before family member’s consumption (not if only index child was eating). For each participant, and for each outcome, the number of times the correct behaviour was performed and the number of opportunities to perform the behaviour during the observation period was recorded. Correct practice of the behaviours, as well as the opportunities to do so, was recorded on a coded structured observations sheet, as used in a previous food and hand hygiene trial.^9^

Other secondary outcomes were: (1) adherence to each individual family-food hygiene behaviour and (2) presence of soap in the kitchen, pit latrine and handwashing station. All outcomes related to the participant level were determined prior to evaluation. An assessment of intervention safety and adverse events was conducted prior to intervention implementation. Participant characteristics were assessed at 6 and 32 months, with village characteristics assessed at baseline. These were cross-sectional surveys, rather than cohort studies; hence, the details of individuals surveyed at each time point varied, as did their summaries.

### Sample size

The sample size for the trial was based on calculations for the 6-month follow-up evaluation of the same intervention, the primary outcome of which was the composite measure of adherence to the five hygiene behaviours when preparing complementary food for 6–36 months old children.^24^ With 15 clusters per arm, at least 12 households per cluster were required to detect a 25% difference in adherence to target complementary-food hygiene behaviours between intervention and control arms (with 95% power, two-sided alpha of 0.05). Unequal cluster sizes were assumed, while an intracluster correlation coefficient of 0.04 and a variation in cluster size coefficient of 0.22 were used. For this study, the sample size was increased to approximately 26 caregivers per cluster, ensuring sufficient numbers of caregivers with children aged 6–24 months (new mothers not pregnant at the time of the intervention) and older children (25–36 months; who were mothers or pregnant at the time of the 4-week campaign) were assessed.

### Analysis

Data were entered into an Excel database and verified against the data entry sheets. For all outcomes, analysis was by intention-to-treat. For all outcomes, three models were run: (1) an unadjusted model, (2) a partially adjusted model (adjusting for cluster level covariates used in stratified randomisation: location north or south of the River Gambia and village size at point of randomisation) and (3) a fully adjusted model (also adjusted for prespecified covariates: caregivers’ age and caregiver’s highest educational attainment). Analyses were performed on the individual level; mixed-effect models were used to offset any variation in outcome between and within villages (clusters). Composite and individual behaviour outcomes were analysed using a mixed-effect Poisson regression model with an offset for the number of opportunities to perform the correct behaviour. Binary outcomes (eg, presence of soap in the household) were analysed using a mixed-effect Poisson regression model with a log-link and robust SEs to obtain risk ratios. Continuous characteristics of the study population at evaluation were assessed for normality using the Shapiro-Wilk test. Descriptive statistics were carried out using SPSS (V.24) while regression models were performed using Stata statistical software, release 15.0 (StataCorp. 2017. Stata Statistical Software: Release 15. College Station, Texas, USA: StataCorp). There were no planned subgroup or sensitivity analyses. No data monitoring committee oversaw the study.

### Role of the funding source

The funders of the study had no role in study design, data collection, data analysis, data interpretation or writing of the report. The corresponding author had full access to all the data in the study and had final responsibility for the decision to submit for publication.

### Patient and public involvement

The public was first involved with the study through mixed methods formative research conducted in three non-study villages in the region.^8^ This helped to identify critical control points of complementary-food contamination (informing outcomes) and investigate motivational drivers of appropriate complementary-food hygiene behaviours (informing the intervention) (online supplemental appendix 3).

Leaders of the included villages (Alkalos) were closely involved with study recruitment and intervention implementation to aid community ‘buy in’ (online supplemental appendix 4). Alkalos were asked to identify traditional birth attendants who were to aid community participation. In congruence with Gambian social norms, village meetings commenced with the local Imam leading community prayers. A video of the Alkalo washing his hands was shown to participants, and in control communities, they distributed vegetable seeds to participant mothers. However, participants were not specifically asked to assess the burden of the intervention and will not be expressly involved in dissemination of study results.

## Results

**Figure 1:**
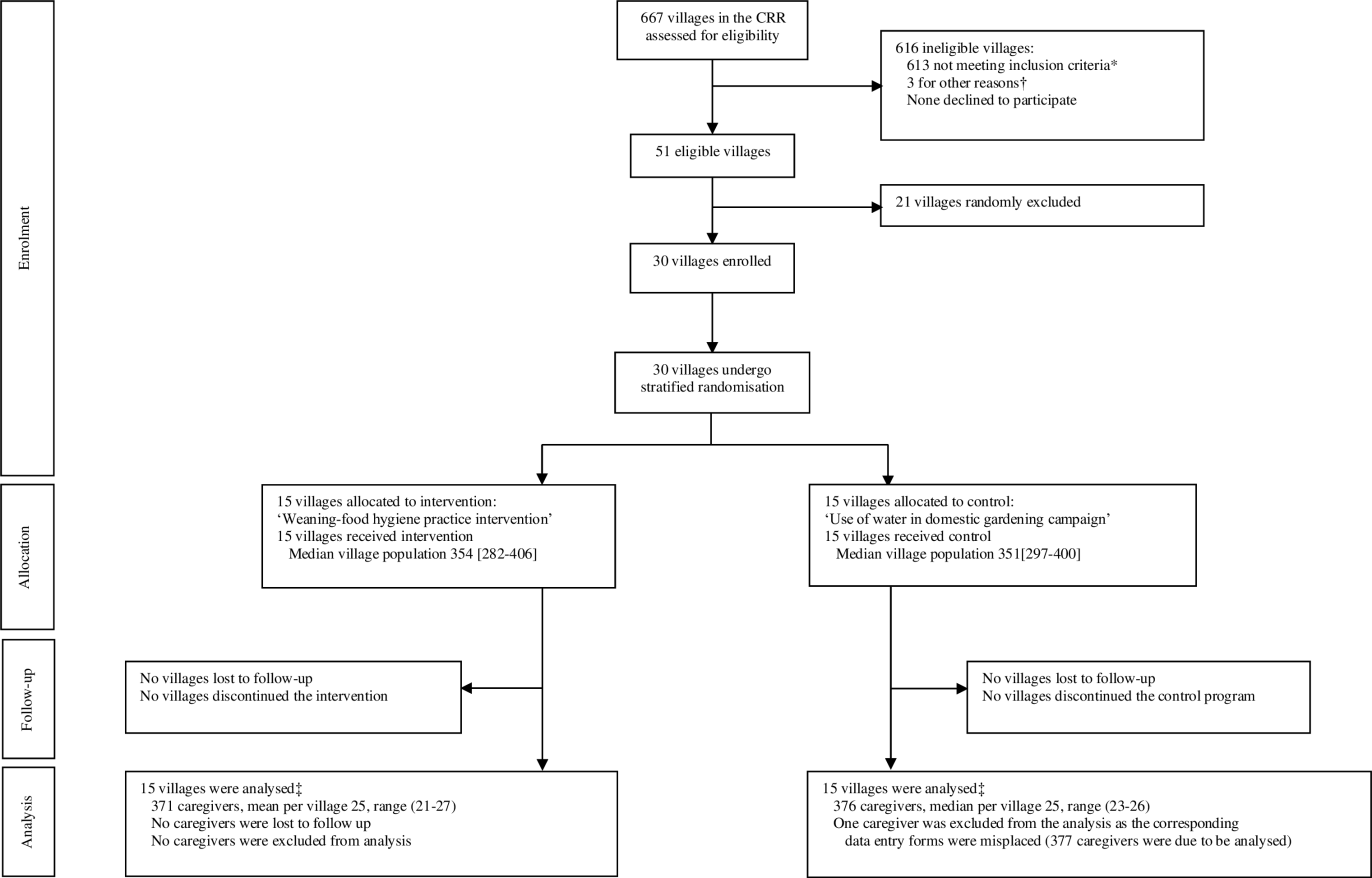
Trial profile. *509 villages were not ‘Primary Healthcare Villages’, 100 villages did not have a population between 200 and 450 and 4 villages were within 5 km of a previously selected village. †3 villages were used for formative research. ‡For the five family food behaviour composite outcome, according to intention-to-treat analysis. CRR, Central River Region.

Between 20 September 2017 and 26 October 2017, 377 caregivers were recruited from 15 control villages, and 371 from 15 intervention villages. One caregiver, allocated to the control group, was excluded from analysis as the data entry forms for the main outcome data were misplaced (figure 1). The vast majority of observed food hygiene behaviours were performed by mothers (82.5%) with the remainder undertaken by other household members (including fathers and grandmothers) who were also exposed to intervention or control programmes (according to village allocation). The sociodemographic characteristics of the caregivers at 32 months (and 6-month assessment of complementary-food hygiene behaviour) were comparable between allocation arms (table 1) as were the characteristics of the villages at baseline.^24^

**Table 1:** Characteristics of the study population at 6-month and 32-month follow-up by intervention allocation

Characteristics	6-month assessment		32-month assessment	
	Control (n=377)	Intervention (n=370)	Control (n=377)	Intervention (n=371)
Age of mother in years, median (IQR)	27 (22–32)	28 (24–32)	26 (22–30)	27 (22–33)
Education of mother				
None/illiterate	148 (48)	138 (45)	233 (62)	206 (56)
Primary	29 (9)	29 (9)	32 (8)	24 (6)
Secondary or higher*	22 (7)	24 (8)	21 (6)	25 (7)
Islamic, incomplete primary or other†	109 (35)	116 (38)	91 (24)	115 (31)
Ethnicity of mother				
Mandinka	71 (23)	89 (29)	60 (16)	93 (25)
Wolof	115 (37)	99 (32)	171 (45)	121 (33)
Fula	122 (40)	119 (39)	138 (37)	151 (41)
Occupation of mother**‡**				
Farmer	279 (91)	285 (93)	307 (81)	312 (84)
Other**§**	29 (9)	22 (7)	70 (19)	58 (16)
Number of children alive for index mother, median (IQR)	3 (2–5)	3 (2–5)	3 (2–5)	4 (2–6)
Belongings				
Land	263 (85)	281 (92)	318 (85)	315 (86)
Cattle	170 (55)	185 (60)	232 (62)	253 (69)
Goat or sheep	242 (79)	247 (80)	313 (83)	345 (94)
Mobile phone	254 (82)	257 (84)	332 (88)	323 (88)
Radio	201 (65)	226 (74)	249 (66)	255 (69)
Water tap	16 (5)	18 (6)	19 (5)	41 (11)
Refrigerator	5 (2)	8 (3)	8 (2)	22 (6)
Source of water				
Covered well	178 (58)	161 (52)	220 (58)	251 (68)
Open well	130 (42)	146 (48)	157 (42)	119 (32)
Structure of house				
Mud wall, corrugated roof	134 (44)	116 (38)	170 (46)	160 (43)
Cement wall, corrugated roof	66 (21)	84 (27)	95 (26)	102 (28)
Mud wall, thatched roof	108 (35)	107 (35)	104 (28)	107 (29)
Pit latrine available	286 (93)	292 (95)	337 (89)	343 (93)

Values are numbers (%) or median (IQR).

* ‘Higher’ consists of college, vocational and university.

† ‘Other’ was French education only.

‡ All mothers may have been housewives, but some had additional responsibilities. 10 mothers stated they were both farmers and involved in animal husbandry or petty trading and are accounted for both in ‘Farmer’ and ‘Other’ categories.

§ Petty trading, civil servant, animal husbandry.

At 32 months, the intervention led to a 17% increase in the practice of the five family-food hygiene behaviours (composite measure): fully adjusted rate ratio (RR) 1.17 (95% CI 1.08 to 1.27, p<0.001 table 2).

**Table 2:** Effect of the intervention on the practice of key family-food preparation behaviours*

Behaviour	Trial arm allocation	Number of correct behaviours	Number of opportunities for performing the correct behaviour	Event rate	Unadjusted RR (95% CI)	Unadjusted p value	Fully adjusted RR (95% CI)	Fully adjusted p value
Five key behaviours	Control	1827	4559	0.40	1.17 (1.07 to 1.28)	<0.001	1.17 (1.08 to 1.27)	<0.001
	Intervention	2073	4425	0.47				
Handwashing before food preparation†	Control	157	604	0.26	1.38 (1.12 to 1.70)	0.002	1.39 (1.12 to 1.72)	0.003
	Intervention	215	599	0.36				
Handwashing when contaminated during cooking†	Control	77	1218	0.06	2.14 (1.63 to 2.81)	<0.001	2.10 (1.58 to 2.80)	<0.001
	Intervention	156	1152	0.14				
Handwashing before eating (caregiver)†	Control	20	708	0.03	3.61 (2.07 to 6.31)	<0.001	3.45 (1.94 to 6.14)	<0.001
	Intervention	70	685	0.10				
Washing of pots and utensils, drying on clean surface‡	Control	1495	1924	0.78	1.06 (0.96 to 1.17)	0.230	1.06 (0.97 to 1.16)	0.184
	Intervention	1549	1885	0.82				
Reheating of food after storage§	Control	78	105	0.74	1.07 (0.79 to 1.46)	0.649	1.06 (0.76 to 1.48)	0.727
	Intervention	83	104	0.80				

* For complete table including crude RR and adjusted RR please see online supplemental appendix 5.

† Handwashing was with water and soap.

‡ Before food preparation or before the caregiver eats.

§ Before the caregiver eats.

RR, rate ratio.

There were improvements in all three handwashing behaviours performed; before food preparation: RR 1.39 (95% CI 1.12 to 1.72, p=0.003), after contamination of hands during cooking: RR 2.10 (95% CI 1.58 to 2.80, p<0.001) and before the caregiver ate: RR 3.45 (95% CI 1.94 to 6.14, p<0.001). Washing of pots and utensils and leaving to dry on a clean surface (RR 1.06, 95% CI 0.97 to 1.16, p=0.184) and reheating of food after storage (RR 1.06, 95% CI 0.76 to 1.48, p=0.727) were adhered to slightly more in the intervention than control caregivers, but differences were not statistically significant.

At 32 months, the intervention led to greater soap availability in all assessed locations: a 16% increase in kitchens (RR 1.16, 95% CI 1.02 to 1.34, p=0.029, table 3), a 32% increase in pit latrines (RR 1.32, 95% CI 1.08 to 1.61, p=0.006) and more than a doubling in other handwashing stations (RR 2.23, 95% CI 1.17 to 4.24, p=0.015). There were no adverse or unintended events.

**Table 3:** Effect of intervention on outcomes other than key food preparation behaviours

Outcome	Control n (%)	Intervention n (%)	Unadjusted RR (95% CI)	Unadjusted p value	Partially adjusted RR (95% CI)	Partially adjusted p value	Fully adjusted RR (95% CI)	Fully adjusted p value
Soap observed in kitchen	236 (63.4)	261 (73.3)	1.16 (1.00 to 1.34)	0.056	1.17 (1.02 to 1.35)	0.028	1.16 (1.02 to 1.34)	0.029
Soap observed in pit latrine	152 (41.1)	198 (55.3)	1.35 (1.08 to 1.68)	0.009	1.38 (1.14 to 1.66)	0.001	1.32 (1.08 to 1.61)	0.006
Soap observed in handwashing station	61 (16.4)	116 (32.4)	2.08 (1.08 to 3.99)	0.028	2.15 (1.15 to 4.00)	0.016	2.23 (1.17 to 4.24)	0.015

RR, relative risk.

## Discussion

We present the results of a 32-month post-intervention evaluation of the MaaChampion cluster-RCT for its additional benefit on improved food hygiene behaviours of caregivers while preparing family food. The MaaChampion intervention improved overall adherence to a composite measure of five behaviours associated with preparing and cooking family food 32 months after intervention delivery. This was mainly driven by improvements in the practice of three behaviours relating to handwashing with soap while preparing food, and was supported by the finding that a greater proportion of the intervention villages had soap for handwashing compared with the control villages.

To the best of our knowledge, this is the first RCT to examine the effect of an intervention on household family-food hygiene practices in LMICs. Our systematic review (publication in draft) highlights five former studies^25^,^29^ that reported gains in knowledge, attitudes and practices concerning family-food hygiene in response to a food hygiene intervention. These studies took place in LMIC settings (Bangladesh, Iran, Cambodia and Laos) and assessed food hygiene interventions of varying complexity from a single small group teaching session in Cambodia and Laos^29^ to eight visits held across 8 months in Bangladesh.^25^ However, studies were generally of poor quality: no trial involved a control group and the majority did not assess the statistical significance of the primary outcome.^2527^,^29^ In all included studies, post-intervention follow-up did not exceed 1 month, and in one study, follow-up took place immediately after intervention delivery^25^, impairing conclusions regarding lasting behaviour change. Furthermore, those delivering the intervention also performed outcome assessment which risked observation bias.^25 28 29^ Encouragingly, intervention development in three sites was informed by the results of a baseline outcome assessment,^27^,^29^ or by formative research in the study site (Bangladesh),^25^ but only one intervention in Bangladesh^25^ targeted emotional drivers of behaviour change (eg, fear or disgust) over ‘Germ Theory’.

Our intervention targeted complementary food but also improved food hygiene behaviours for the wider family that was still evident 32 months later. Improvements in complementary-food hygiene behaviours at 6 months more pronounced than at 32 months follow-up;^24^ hence, we would expect the same for family-food hygiene behaviours at 6 months (although this was not assessed) with possible health benefit to family members. At 32 months, the intervention significantly improved three handwashing behaviours among five tested family-food hygiene practices. Internal validity is provided through increased soap availability noted in intervention homes, despite no soap distribution or any messages to enforce having soap in the kitchen. This may have been due to particularly effective intervention components that specifically addressed food-hygiene related handwashing: the ‘Glow-Germ’ game and/or the ‘SuperAmma’ handwashing animation videos.^17^ Alternatively, handwashing-related interventions by other organisations (including non-governmental organisations) in the region since our intervention may have inadvertently had a greater impact among our intervention villages over those allocated to the control group.

The intervention did not sustain benefits in reheating leftover food for family consumption, although the study found limited opportunities for reheating family food in both intervention and control groups as both tended to cook fresh dishes for adult meals, likely reducing the power to detect any differences. In The Gambia, formative research indicated that adult leftovers were usually reheated before eating anyway (not commonly done for child’s complementary food).^24^ Differences in the practice of washing pots and utensils were minimal between groups. At the 6-month assessment, this behaviour, regarding complementary food, was uncommon only in control villages^24^; similar findings were observed during formative research.^8^ By 32 months, adherence to this practice was high (around 80%) among intervention and control groups regarding complementary food, attributed to cross-contamination.^24^ Additionally, qualitative data suggested that villagers took pride in sharing adopted behaviours with others as MaaChampions and MaaSupervisors,^24^ and as washing pots and drying on a clean surface was the most visible behaviour to outsiders, this may have been adopted more than handwashing behaviours, affecting both child and family food preparations similarly.

Globally, children under 5 years of age have the greatest rates of diarrhoea morbidity of any age group^30^ and this intervention reduced diarrhoea among children aged 6–36 months.^24^ This was probably because, in addition to poor complementary-food hygiene practices, poor family-food hygiene behaviour also places children under 5 years of age at risk of diarrhoea, as family food is also fed to the majority of children aged 18 months to 5 years in LMICs,^8^,^13^ with children aged 6–24 months being frequently fed family food in West Africa.^10^ Additionally, complementary food is at risk of contamination from poor family food hygiene practice as caregivers in The Gambia may prepare both foods simultaneously.^8^ Therefore, improvements in handwashing behaviour when preparing family food, as well as subsequent reduced diarrhoea in family members resulting in a reduced environmental prevalence of pathogens, may have contributed to the reduction in diarrhoea incidence among complementary food aged children as previously reported.^24^ Furthermore, the importance of the reduction of diarrhoeal disease among those aged over 5 years must not be overlooked. Globally, diarrhoeal diseases remain among the top 12 causes for disability adjusted life years among adolescents, young adults and the elderly.^31^

Strengths of this study include the intervention’s strong theoretical base, previously used in other successful food and hand hygiene trials.^9 17^ Powerful motivational drivers were strongly associated with the target behaviours in the stories, drama and songs. The intervention also prepared the social environment by gaining community support through the involvement of key figures like older mothers, fathers and elders, and by encouraging caregivers to publicly commit to adopting the behaviours. These actions aimed to elevate their status as role models, supported by displaying their photos, offering peer education and mobilising the community.^23 32^ Qualitative data indicate that the MaaChampion programme was seen as a beneficial and joyful local initiative, visibly improving children’s health and reducing family expenses. The physical environment saw minimal change, except for the plastic sheets that were distributed for drying dishes, placing educational posters and danglers in kitchens and rewarding successful caregivers with a bar of soap. The intervention was low-cost,^24 33^ culturally sensitive, used local healthcare workers and was based on mixed methods formative research in the region. These factors have been called for by Woldt and Moy^5^, Touré *et al* and Curtis *et al*^22 34^ to encourage intervention delivery and behaviour uptake at scale.

Regarding outcome assessment, structured observation sheets were used to assess food hygiene behaviours, rather than self-reported questionnaires, as the latter frequently over-report the incidence of behaviours.^35^ Moreover, the period of daily observing caregivers was more than twice as long as previous community-based hygiene trials,^9 17 36^ with family-food preparation being assessed at both breakfast and lunchtime, improving the reliability of the findings. Overall, this is the longest follow-up of any food hygiene intervention conducted in a domestic LMIC setting to date, thus addressing the question of whether behavioural improvements are sustained in the long term.

Limitations include the possible presence of reactivity and observation bias.^24^ Reactivity bias occurs where caregivers may have only demonstrated improved food hygiene practice because they were aware they were being observed.^37^ However, the underlying purpose of the data collection was concealed from participants and fieldworkers, and we attempted to minimise this bias by only having two follow-ups and not evaluating food preparation repeatedly.^17^ We also used different teams to deliver the intervention, collect baseline and outcome measurements. The follow-up time meant that the majority of children at the 6-month follow-up were not in the age range at the 32-month follow-up and together with random selection helped to reduce the chance of the same caregivers being assessed at both 6 and 32 months. Outcome evaluation took place in one village per day, ensuring caregivers were not able to discuss the fieldworker observations and questions among themselves between assessment days. There is likely to have been intervention cross-contamination, whereby control caregivers may have adopted family-food hygiene behaviours from intervention villages by the time of 32-month follow-up, which may have diluted effect sizes.^24^

Observer bias was curtailed in that outcome assessors were not made aware of the intervention, nor of the purpose of the evaluation, and were recruited from non-study villages. Nevertheless, participant caregivers may have informed observers they had MaaChampion in their village, which may have influenced the observer’s assessment of behaviour. It was also not possible to establish whether an increase in handwashing with soap led to a direct reduction in microbiological contamination of family food, as we were unable to measure faecal form counts. However, at 6-month follow-up,^24^ reductions in laboratory assessed *Escherichia coli* counts of complementary-food samples were statistically significant and mirrored the statistical significance of behaviour changes observed in the mother’s adherence to complementary-food hygiene behaviours. It is therefore conceivable that the improved adherence to family-food hygiene behaviours we observed also improved microbiological contamination rates of family-food.

In summary, the MaaChampion cluster-RCT found significant improvements in caregiver’s family-food handwashing practices nearly 2.5 years following an intervention to improve their complementary-food hygiene practices, without further community visits. This addressed the clear evidence gap we have identified in the literature.^25^,^29^ It is significant that our study’s outcome behaviours were not the primary objective of the intervention and were observed with important methodological improvements to reduce reactivity and observation biases. This could have important implications for adults and children over 2 years of age, thus reducing the pool of infection and cases of diarrhoea morbidity and mortality in all age groups. Therefore, this intervention warrants replication and testing in larger trials^38^ which ought to target both complementary-food and family-food hygiene behaviours.

## Supplementary material

10.1136/bmjgh-2024-017026 online supplemental file 1

10.1136/bmjgh-2024-017026 online supplemental file 2

## Data Availability

Data are available upon reasonable request.
