# Supplementary material for: A community-level complementary-food safety and hygiene intervention improves family-food preparation behaviours in rural Gambia: a follow-up of a cluster randomised controlled trial
Source: BMJ Glob Health. 2026 Mar 18;11(3):e017026. doi: 10.1136/bmjgh-2024-017026 (PMC13034271; doi:10.1136/bmjgh-2024-017026)
Supplement: online supplemental file 1 [file bmjgh-11-3-s001.pdf]

Appendix

Appendix 1: Trial Timeline……………………………………………………………………………………………………...2

Appendix 2: Precautions Used to Minimise Observer and Reaction Bias……………………………………………………..3

Appendix 3: Summary of the Intervention Design…………………………………………………………………………...4

Appendix 4: Details of Daily Intervention Activities……………………………………………………………………….....5

Appendix 5: Effect of the Intervention on the Practice of Key Family-food Preparation Behaviours……………………………..8

Appendix 1: Trial Timeline

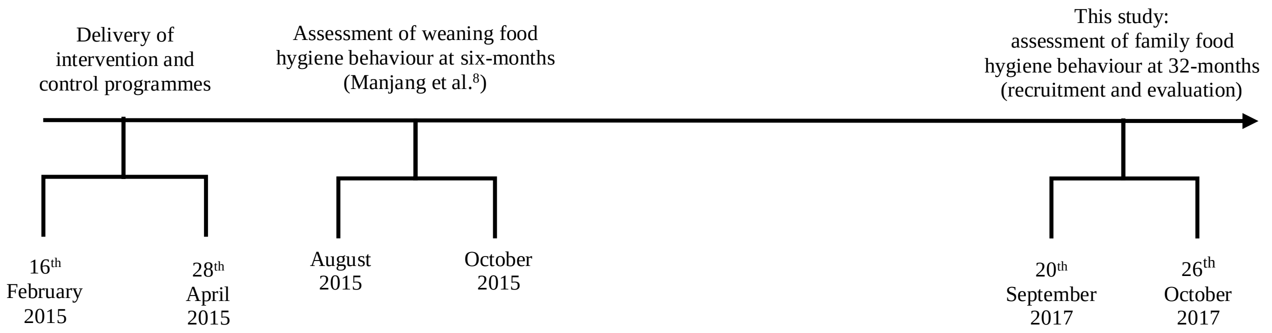

Appendix 2: Precautions Used to Minimise Observer and Reaction Bias*

Bias reduced		Measures Taken
**Reactivity bias in mother (RB)**	**Observation bias in data-collectors (OB)**	
RB		Reduction of the study population's exposure to the trial procedures (using random cross-sectional samples to observe mothers' behaviours on only 3 occasions, baseline, 6- and 32-months) [15-17].
RB		Using different research teams and methods for baseline and the assessment rounds: for baseline, male researchers surveying the mother with a short questionnaire at the door, and different teams of female data-collectors recording same questionnaire responses as a part of a longer assessment questionnaire during the 9-hour home-visit observation and survey [15-17].
RB		Ensuring the 6- and 32-month assessment teams stayed only one day in each village [9], and thus mothers were unable to discuss data collection procedures between data collection days.
RB	OB	Concealment of the purpose of 6- and 32-month assessments from mothers and data-collectors by conducting a larger assessment of water and food utilisation in households and health related measures. This was facilitated as follows:<br>• During training and consent from mothers the survey/observations were described as a larger assessment of water and food utilisation in households and health related measures.<br>• Not informing the data-collectors that an intervention and a trial had taken place.<br>• Concealment of complementary-food assessment tools within a larger assessment of food and water usage observation and questionnaire tools. At 32-months additional questions and formats related to observing the child behaviour/play, ARI health economics, and details of water sources were added to the 6-month tool.<br>• Consenting the mothers for this larger assessment and not for the complementary-food safety and hygiene trial.<br>• Concealment from mothers and data-collectors was aided by the fact that other government, NGOs, and UN agencies and MRC Gambia have numerous concurrent nutrition/WaSH intervention programmes, studies and surveillance sites in this region resulting in numerous visits to villages for interventions and data collection. In terms of intervention material in the villages, where posters or village banners related to complementary-food safety and hygiene remained, these would have competed with other intervention activity posters and banners related to other agency programmes which are prevalent in rural LMIC communities.
OB		No contact between the study or intervention team and the villages between 6- and 32-month follow-up. This meant that at 32-months, both control and intervention villages were equally unlikely to link the 32-month assessment with the complementary-food and hygiene programme having not had any reminders or contact for over 2 years and that the families were mostly 'new mothers'.

* Manaseki-Holland S, Manjang B, Hemming K, Martin JT, Bradley C, Jackson L et al. Effects on childhood infections of promoting safe and hygienic complementary-food handling practices through a community-based programme: A cluster randomised controlled trial in a rural area of The Gambia. PLoS Med 2021 2021 Jan 11; **18**(1): 1003260.

Appendix 3: Summary of the Intervention Design

Prioritised CCPs after a HACCP analysis, conducted during formative research, identified a range of CCPs: *

1. Unwashed hands before food preparation
2. Unwashed hands during cooking when hands become contaminated
3. Use of inadequately washed utensils or washed utensils dried on an unclean surface
4. Unwashed hands before feeding the child
5. Prolonged inappropriate storage without reheating before feeding to the child
6. Lack of clean water ready to drink by the child

Six complementary-food-safety and hygiene behaviours (corrective measures for above mentioned prioritised CCPs): *

1. Handwashing with soap and water before food preparation
2. Handwashing with soap and water when contaminated during cooking
3. Handwashing with soap and water before feeding child (caregiver) or eating (child)
4. Washing of pots and utensils before food preparation and drying on a clean surface
5. Reheating of pre-made food after storage before feeding
6. Boiling of water ready for drinking (child)

Evo-Eco model motivational drivers for handwashing behaviour change: *

1. Nurture **-** the desire for a happy, thriving child
2. Affiliation **-** the desire to fit in with what others in a reference group are doing
3. Disgust **-** the desire to avoid and remove contamination
4. Status **-** the desire to have greater status than others in the group and enjoy a higher respect
5. Purity **-** the desire to be favoured by God and to be holy

Cost:†
The cost of material production and implementation for the intervention in 15 villages over eight months was £13,299 ($US 2014). This is equal to £23·28 per child aged six-24 months or £2·55 per person.

***** Manjang B, Hemming K, Bradley C, Ensink J, Martin JT, Sowe J, et al. Promoting hygienic weaning food handling practices through a community-based programme: Intervention implementation and baseline characteristics for a cluster randomised controlled trial in rural Gambia. *BMJ Open* 2018; **8**.

† Manjang B. Investigating effectiveness of behavioural change intervention in improving mothers weaning food handling practices: design of a cluster randomized controlled trial in rural Gambia [PhD thesis]. Birmingham (UK): University of Birmingham; 2016.

Appendix 4: Details of Daily Intervention Activities*

Day 1

Event	Activity	Location	Time	Purpose
Meeting the Alkalo (village head)	- TCs play a song in praise of the Alkalo.<br>- The team greet the Alkalo<br>- Explain purpose/project<br>- Meet VHW & TBA and MaaSupervisor	Alkalo's residence	20 min	- Alkalo is the entry point to the village; must receive a visit before start of work<br>- Alkalo & wife have social status & their support motivates mothers
Announce to the villagers	-TCs invite villagers to afternoon meeting by drumming & campaign song with the use of a loud speaker	Within whole village	2 hrs	- Create alert<br>- Mobilise the community<br>- Memorisation assisted by repetition of song & messages
House-to-house visit with MaaSupervisors	- House-to-house visit (invite household members to afternoon meeting) with TBA & VHW	Residence of every household especially with young children	3 hrs	- Social mobilisation to involve the whole community
Record a short video	- Video the Alkalo & wife handwashing & reheating complementary-food to show at the village meeting later	Alkalo's residence	15 min	- Alkalo & wife have social status & their support motivates mothers -Engender a social norm
Afternoon event	-TC's Drum/sing the 6 messages & pledging song while villagers arrive at meeting site<br>- Opening prayers by the Imam (religious leader) lead prayers for the gathering (Gambian cultural norm)<br>- Opening remark by the Alkalo.<br>- Introduction of project by PHO<br>- 2 Drama (MaaChampion & Funtu) by TCs<br>- Summary of 6 messages from the drama by PHO.<br>- Question & answer from village audience led by PHO<br>- Pledge song by TCs<br>- Play 'Choose soap' silent animation video.<br>- Show video of Alkalo (washing hands with soap) & his wife (reheated complementary-food) translated live in local language<br>- Announce MaaChampion competition by PHO.<br>- Invite mothers of children 6-24m to pledge to practise behaviours<br>- Give pledged mother's plastic sheets for covering surfaces to enable hygienic drying of utensils/pots on a clean surface<br>- Take a group photo of pledged mothers for the honour board.<br>- Closing remark by PHO.<br>- More drumming & songs (motivational). - Print & display pledged mothers & Alkalo's photos on honour board at the Bantaba	Village "Bantaba" (a central place where villagers meets – usually under a large tree)	4 hrs	-Inform the community/provide instructions<br>- Model or demonstrate behaviour<br>- Engender all motivational drivers, particularly nurture and affiliation<br>- Prompt identification with a role model<br>- Prompt action through pledging<br>- Set graded tasks through competitions<br>- Target mothers for their pledge<br>- Prompt intention formation<br>- Memorisation assisted by repetition of song & messages<br>- Display of photos of pledged mothers for contingent reward
Community volunteers training	- Train new assistant MaaSupervisors by village volunteers/trained MaaSupervisors, supervised by PHO	Village Bantaba	2 hrs	- Enable encouragement of mothers and competition success<br>- Involve more community members to engender development of social norms

Day 2

Event	Activity	Location	Time	Purpose
Meeting the Alkalo	- Greet Alkalo as Day 1	Alkalo's residence	10 min	As Day 1
Announce to the villagers	As Day 1	As Day 1	2 hrs	As Day 1
House-to-house visit with Maa-Supervisors	- Engage MaaSupervisors with household visits & boost their confidence<br>- Assess/encourage pledged mothers for progress to next stage	Residence of each pledged mother	3 hrs	- Prompt practice of key behaviours<br>- Provide feedback<br>- Prompt self-monitoring /review/community mobilisation
Ad-hoc women or men meetings held separately in neighbour-hoods	- Glow germ demonstration<br>- Explain 2 stories (MaaChampion & Funtu) on flipchart<br>- Play silent animation video 'Choose soap' on iPad/laptop<br>- Visit home of mothers with the MaaSupervisors to assess them for MaaChampion status	Neighbour-hoods	30 min	- Engender disgust through glow-germ: dirt on hands<br>- Engender all motivational drivers – particularly nurture and affiliation through stories<br>- Engage men & women to support mothers of young children<br>- Prompt specific goal setting

Day 3

Event	Activity	Location	Time	Purpose
Meeting Alkalo	As Day 2	As Day 2	20 min	As Day 2
Announce to the villagers	As Day 1	As Day 1	2 hrs	As Day 1
House-to-house visit with MaaSupervisors	As Day 2 – additionally:<br>- During household visits, video mothers who succeeded to became a MaaChampion to show at meetings	As Day 2	3 hrs	As Day 2 – additionally videoing to provide contingent reward
Afternoon event	As Day 1 & including the below:<br>- Show animation video from India handwashing "SuperAmma" project with spontaneous translation[10]<br>- Show videos from mothers who succeeded to become MaaChampions<br>- Take photo of new pledged mothers with their plastic sheets and of MaaChampion's with medals & displayed on honour boards (pledging and certification ceremonies)	As Day 1	4 hrs	As Day 1

Day 4

Event	Activity	Location	Time	Purpose
Meeting Alkalo	As Day 2	As Day 2	20 min	As Day 2
Announce to village	As Day 1	As Day 1	2 hrs	As Day 1
House-to-house visit with MaaSupervisors	As Day 3	As Day 3	3 hrs	As Day 3
Afternoon Event	As Day 1 including below:<br>- Certification ceremony: Present medals for MaaChampions / MaaSupervisors with drumming<br>- Group picture with all MaaChampions, MaaSawarr and MaaFamboos for the honour board<br>- During village wide ceremony, erect a complementary-food hygiene board at the village entrance establishing the village as a "complementary-food hygiene" village with drumming/campaign songs & present village certificate to the Alkalo (Certification ceremony)<br>- Give motivational advice on sustainability by Alkalo, MaaSupervisors,PHOs<br>- Closing remark (emphasis on sustainability)	As Day 1	4 hrs	As Day 1 including below:<br>- Create ownership of the project and self-monitoring to enable sustainability<br>- A community sense of achievement, and pride commitment by MaaChampions<br>- Inculcated motivational drivers<br>- Encourage achievement of goals through the board as a reminder

Day 5

Event	Activity	Location	Time	Purpose
Meeting Alkalo	As Day 2	As Day 2	20 min	As Day 2
Announce to the villagers	As Day 1	As Day 1	2 hrs	As Day 1
House-to-house visit with MaaSupervisors	As Day 3	As Day 3	3 hrs	As Day 3
Afternoon event	As Day 4 but not including erection of the village board or certification	As Day 4	4 hrs	As Day 4

TC= Traditional Communicator, PHO= Public Health Officer, VHW=Village Health Volunteer, TBA=Traditional Birth Attendant, Alkalo = village head, MaaChampion = role model described in intervention (status achievable by mothers), Funtu = unpopular character who did not practice the family-food hygiene behaviours, MaaSawarr = mothers who had sustained practice of the five family-food hygiene behaviours , MaaFamboos = mothers who had pledged to adopt the five family-food hygiene behaviours, MaaSupervisors = assistant volunteer mothers, recruited by village TBAs (used to encourage adherence amongst mothers), Banataba = village central meeting place.

***** Manjang B, Hemming K, Bradley C, Ensink J, Martin JT, Sowe J, et al. Promoting hygienic weaning food handling practices through a community-based programme: Intervention implementation and baseline characteristics for a cluster randomised controlled trial in rural Gambia. *BMJ Open* 2018; **8**.

Appendix 5: Effect of the Intervention on the Practice of Key Family-food Preparation Behaviours

Behaviour	Trial Arm Allocation	Number of Correct Behaviours	Number of Opportunities for Performing the Correct Behaviour	Event Rate	Crude RR	Unadjusted RR (95% CI)	Unadjusted p value	Adjusted RR (95% CI)	Adjusted p Value	Fully Adjusted RR (95% CI)	Fully Adjusted p value
**Five Key Behaviours**	Control	1827	4559	0·40	1·17	1·17 (1·07 to 1·28)	<0·001	1·18 (1·08 to 1·28)	<0·001	1·17 (1·08 to 1·27)	<0·001
	Intervention	2073	4425	0·47							
**Handwashing Before Food Preparation***	Control	157	604	0·26	1·38	1·38 (1·12 to 1·70)	0·002	1·42 (1·16 to 1·75)	0·001	1·39 (1·12 to 1·72)	0·003
	Intervention	215	599	0·36							
**Handwashing When Contaminated During Cooking***	Control	77	1218	0·06	2·14	2·14 (1·63 to 2·81)	<0·001	2·13 (1·62 to 2·80)	<0·001	2·10 (1·58 to 2·80)	<0·001
	Intervention	156	1152	0·14							
**Handwashing Before Eating (Caregiver)***	Control	20	708	0·03	3·62	3·61 (2·07 to 6·31)	<0·001	3·75 (2·14 to 6·57)	<0·001	3·45 (1·94 to 6·14)	<0·001
	Intervention	70	685	0·10							
**Washing of Pots and Utensils, drying on clean surface†**	Control	1495	1924	0·78	1.06	1·06 (0·96 to 1·17)	0·230	1·06 (0·97 to 1·17)	0·204	1·06 (0·97 to 1·16)	0·184
	Intervention	1549	1885	0·82							
**Reheating of Food After Storage‡**	Control	78	105	0·74	1·07	1·07 (0·79 to 1·46)	0·649	1·06 (0·78 to 1·45)	0·693	1·06 (0·76 to 1·48)	0·727
	Intervention	83	104	0·80							

*RR = Rate Ratio *Handwashing was with water and soap. †Before food preparation or before the caregiver eats. ‡Before the caregiver eats*
