# Supplementary material for: A community-level complementary-food safety and hygiene intervention improves family-food preparation behaviours in rural Gambia: a follow-up of a cluster randomised controlled trial
Source: BMJ Glob Health. 2026 Mar 18;11(3):e017026. doi: 10.1136/bmjgh-2024-017026 (PMC13034271; doi:10.1136/bmjgh-2024-017026)
Supplement: online supplemental file 2 [file bmjgh-11-3-s002.pdf]

Author Reflexivity Statement

1. **How does this study address local research and policy priorities?**

This study is a 32-month follow-up of a community food hygiene intervention that was central to BM's PhD thesis for the University of Birmingham. BM is a Gambian national who has since gone on to become deputy Director for Public Health Services of The Gambia and has thereby taken his experience oof intervention implementation into his ongoing work to improve public health in The Gambia. This study helps to address the key priorities identified by BM as it establishes the indirect effects of his intervention on improving family food hygiene practices, which may in turn improve infant and child health as family food is often fed to children under five years old in The Gambia. The results have been reported back to Ministry of Health and national nutrition agencies who were partners and co-funders (in-kind) for this study.

2. **How were local researchers involved in study design?**

As mentioned, BM was instrumental in designing and overseeing implementation of the intervention. Although no other co-authors were from low-income countries, SMH and SC have previously led on numerous studies set in low and low-middle income nations aiming to improve maternal child health and environmental health engineering respectively. For other parts of the RCT other significant contributors have been co-authors in publications from the national lab (data not used here), or nutrition agency (qualitative data not used here).

3. **How has funding been used to support the local research team?**

Funding from the SHARE consortium and UNICEF funded the initial fieldwork related to the intervention, in addition to the Islamic Development Bank which provided scholarship for BM's PhD. Funding from the MRC and Ministry of Health of The Gambia facilitated fieldwork for the 32-month follow up. This project had almost no funds spent in the UK since the first author was a UK medical student who funded his own trip.

4. **How are research staff who conducted data collection acknowledged?**

Key study fieldworkers are explicitly referenced and thanked in the acknowledgement section.

5. **Do all members of the research partnership have access to study data?**

All members of the partnership have access to the study data.

6. **How was data used to develop analytical skills within the partnership?**

BM analysed the first stage of the RCT published in Plos Med. He was too occupied as in his work for the Ministry of Health to perform further analysis for this publication. Given that no more resources were available to employ anyone from The Gambia, WH's assistance was sought as first author. WH's data

analytical skills were developed through direct teaching and supervision by JM (lead statistician on the project) and manuscript composition skills though SMH's supervision. Data cleaning was performed by WH and EHS with guidance provided by SMH.

7. How have research partners collaborated in interpreting study data?

BM was not directly involved with interpreting the 32-month follow up data relating to family-food, rather the 6-month follow up which was published separately. Data interpretation was performed jointly by WH, SMH and FC, however the results were extensively discussed within the wider authorship who have collectively agreed on the results and conclusions.

8. How were research partners supported to develop writing skills?

The intervention this study describes was in part implemented by BM as part of his doctoral thesis at the University of Birmingham during which he was supported in his writing skills by his supervisor, SMH, and the wider post-graduate team. SMH and FC also helped to develop the writing skills of WH, an early career researcher, through supervision of his drafting of the manuscript.

9. How will research products be shared to address local needs?

Once published, the study results will be relayed to the Public Health Services Department of The Gambia, helping them to identify effective components of successful behavioural change interventions informing their efforts to improve food hygiene practices across the country.

10. How is the leadership, contribution and ownership of this work by LMIC researchers recognised within the authorship?

The PhD thesis of BM is foundational to this paper: encompassing its conceptualisation, study design, intervention development and implementation. However, while BM managed the collection of data for the 32-month follow up, the data verification, analysis and manuscript compilation was performed by other co-authors as BM was too occupied to contribute to this. However, we believe the second author position remains significant. We had intended to include more LMIC laboratory partners as co-authors, including Bakary Sanneh (Principal Laboratory Scientist of the National Public Health Laboratories of The Gambia). However, we were not successful in obtaining coliform contamination rates of family food samples from study villages, so we were unable to proceed with this outcome.

11. How have early career researchers across the partnership been included within the authorship team?

Two early careers researchers, admittedly from high-income countries, performed data analysis (WH and EHS) with WH also drafting the manuscript and assisting with data verification with supervision from the

senior authors. From low-income country settings BM and local Public Health Officers (see below) were also early career researchers.

12. How has gender balance been addressed within the authorship?

The gender balance within the authorship is relatively equal. Of the seven authors four are male (WH,BM, JM, SC) and three female (SMH,FC, EHS): two of whom are classed as senior authors.

13. How has the project contributed to training of LMIC researchers?

As mentioned, the study formed part of PhD thesis of BM who was at the time a mid-grade public health officer in a remote Gambian province. Within two years of graduation from his PhD he became the Deputy Director of Public Health for The Gambia. He has therefore been able to guide the department's research agenda based on his PhD experience, and continues to lecture in food hygiene at the University of Banjul. In addition, fieldworkers, including three Public Health Officers from the local Regional Public Health Department, received significant research training in methodology and data collection during project implementation.

14. How has the project contributed to improvements in local infrastructure?

Various environmental cues were distributed to intervention households including food hygiene posters and danglers to be used around the home environment, as well as plastic sheeting to store washed dishes on. These were left for household use following the intervention. Furthermore, during a village wide ceremony a permanent sign was erected at the entrance to each intervention village, identifying it as a weaning food hygiene village.

15. What safeguarding procedures were used to protect local study participants and researchers?

There were no high-risk situations to require specific safeguarding measures at the time of the study. Caregivers (typically mothers) were interviewed by female researchers who were often younger than them. No other contacts with participants were made.
